# Microstructure and Deformation Response of TRIP-Steel Syntactic Foams to Quasi-Static and Dynamic Compressive Loads

**DOI:** 10.3390/ma11050656

**Published:** 2018-04-24

**Authors:** David Ehinger, Jörg Weise, Joachim Baumeister, Alexander Funk, Anja Waske, Lutz Krüger, Ulrich Martin

**Affiliations:** 1 Leibniz Institute for Solid State and Materials Research IFW Dresden, Institute for Complex Materials, Helmholtzstr, 20, D-01069 Dresden, Germany; David.Ehinger@filkfreiberg.de (D.E.); a.funk@ifw-dresden.de (A.F.); a.waske@ifw-dresden.de (A.W.); 2 Research Institute of Leather and Plastic Sheeting (FILK) gGmbH, Meißner Ring 1-5, D-09599 Freiberg, Germany; 3 Fraunhofer Institute for Manufacturing Technology and Advanced Materials, Wiener Str. 12, D-28359 Bremen, Germany; Joerg.Weise@ifam.fraunhofer.de; 4 Institute of Materials Engineering, Technische Universität Bergakademie Freiberg, Gustav-Zeuner-Str. 5, D-09599 Freiberg, Germany; Krueger@ww.tu-freiberg.de (L.K.); martin@ww.tu-freiberg.d (U.M.)

**Keywords:** syntactic foams, steel matrix, TRIP, martensite, high strain rate, quasi-adiabatic

## Abstract

The implementation of hollow S60HS glass microspheres and Fillite 106 cenospheres in a martensitically transformable AISI 304L stainless steel matrix was realized by means of metal injection molding of feedstock with varying fractions of the filler material. The so-called TRIP-steel syntactic foams were studied with respect to their behavior under quasi-static compression and dynamic impact loading. The interplay between matrix material behavior and foam structure was discussed in relation to the findings of micro-structural investigations, electron back scatter diffraction EBSD phase analyses and magnetic measurements. During processing, the cenospheres remained relatively stable retaining their shape while the glass microspheres underwent disintegration associated with the formation of pre-cracked irregular inclusions. Consequently, the AISI 304L/Fillite 106 syntactic foams exhibited a higher compression stress level and energy absorption capability as compared to the S60HS-containing variants. The $\alpha^{\prime }$ -martensite kinetic of the steel matrix was significantly influenced by material composition, strain rate and arising deformation temperature. The highest ferromagnetic $\alpha^{\prime }$-martensite phase fraction was detected for the AISI 304L/S60HS batches and the lowest for the TRIP-steel bulk material. Quasi-adiabatic sample heating, a gradual decrease in strain rate and an enhanced degree of damage controlled the mechanical deformation response of the studied syntactic foams under dynamic impact loading.

## 1. Introduction

Syntactic foams form a subgroup of cellular solids and consist of hollow glass, carbon or ceramic microspheres (microballoons) acting as porosity-producing agents and simultaneously as stiff reinforcements homogeneously distributed in a continuous matrix, as well-known from particulate composites [1,2,3]. Metal matrix syntactic foams (MMSFs) mostly superimpose their cellular metallic opponents by their pronounced average strength and energy absorption capability under compressive and even tensile loading [4,5]. The most common methods for synthesis, which have been already applied to Al-, Zn-, Mg-, Ti-, Ni-, Fe-based materials, steels and amorphous alloys, are liquid metal processing including pressure infiltration, die casting, stir casting (also in combination with hot extrusion), gravity-fed infiltration, and powder metallurgy routes (e.g., metal powder injection molding (MIM)) [6,7]. The latter is very beneficial since the range of microsphere volume fractions can be widely varied and also matrix compositions, which are not suited for liquid metal processing, as well as high-melting matrices can be used [3].

Besides their good uniaxial mechanical response, generally, the MMSFs offer a reduced thermal conductivity and thermal expansion coefficient, a high energy damping capacity and a distinctive wear resistance [8,9,10,11] that is why they are interesting for passenger safety applications, protecting systems on civil defense and military vehicles, for packaging but also for acoustic damping panels and electromagnetic shielding [3,12,13,14]. Particularly with regard to energy-absorbing structures, Fe-based or steel matrix syntactic foams should be favorable profiting from their high yield strength and more or less pronounced work hardening behavior. Up to now there are only few publications focusing on these materials. Glass microspheres of type iM30K or S60HS (soda-lime borosilicate glass), alumina spheres and cenospheres (fly ash from coal combustion process) were typically used as secondary components [5,15,16,17,18,19].

The stress-strain response of the MMSFs is mostly controlled by the alloy composition (and heat treatment) of the matrix material, the particles’ type and volume content. Further influencing factors are the size (shell thickness-to-diameter ratio) and the size distribution of the spheres as well as the test temperature and applied strain rate. As already known from previous publications, the increase in strain rate can affect the behavior of a cellular composite structure in different ways, including the strain rate sensitivity of the matrix, constraint effects corresponding to the particles, the compression of entrapped fluid, micro-inertia mechanisms and plastic or shock wave propagation [20]. Hence, depending on the combined material components and the applied decade of strain rate different deformation and failure behaviors of the syntactic foams can be expected [1,14,16,21,22,23,24,25,26,27,28]. In the case of the MMSFs based on Fe99.7 [16] and the austenitic stainless steel AISI 316L [29], respectively, an increase in average compression stress with rising strain rate was proven. However, the strain rate sensitivity is not significantly influenced by filler content.

The present study was focused on the quasi-static and dynamic compressive response of syntactic foams with hollow spheres of different type and volume fraction embedded in an austenitic stainless steel matrix. The used CrNi-steel AISI 304L reveals a pronounced strain hardening behavior under mechanical loading contributed by a deformation-induced martensitic phase transformation, also known as TRansformation Induced Plasticity (TRIP) effect. Complex geometric structures such as syntactic foams imply local stress and strain concentrations during plastic deformation. By using either S60HS glass microspheres or Fillite 106 cenospheres as secondary component, differences in stress-strain behavior and deformation mechanisms due to their unequal thermal and mechanical stabilities (see Table 1) were expected.

Altogether, the specific matrix material behavior and structural response can cause an interesting interplay ([30,31,32,33,34]). The aim of the present study is to investigate the interplay between the strain-induced martensitic phase transformation of this steel and the complex structure of the syntactic foam.

Microstructure characteristics, damage evolution and martensitic phase transformation were investigated by X-ray computed tomography measurements (XCT), SEM and EBSD analyses. Magnetization measurements were applied in order to determine the fraction of the ferromagnetic $\alpha^{\prime }$-martensite phase and its kinetic as a function of material condition, deformation degree and strain rate.

## 2. Materials and Methods 

### 2.1. Materials and Processing

The syntactic TRIP-steel matrix foams were fabricated by means of metal powder injection molding (MIM) according to the procedure as described by [5,19]. First, the MIM-feedstock was prepared by mixing the components of the binder and the metal powder plus hollow spheres to a volume ratio of 2:3. The metallic constituent used as matrix was given by the stainless steel powder AT304L-PF10 (0.024% C, 18.9% Cr, 9.6% Ni, 0.3% Si) from Epson Atmix Corporation (Hashinohe, Japan) with a mean particle size of 6 µm. Soda-lime borosilicate 3M^TM^ S60HS glass hollow spheres (3M Deutschland GmbH, D-41453 Neuss, Germany) or Omya Fillite^®^ FG 106 alumino-silicate cenospheres (Omya GmbH, D-50679 Köln, Germany) were added in varying quantities offering different batches of material with 0% (viz. 100% steel specimens; as reference material), 20%, 40% and 60% spheres per volume. Certain properties of the hollow sphere materials obtained from the datasheets of the suppliers [35,36] are listed in Table 1. In order to give a better insight into the structure-material-relationships, the test specimens ranged from bulk (or porous solid) to cellular state, whereby, the last complies with the definition of foam. The used material fractions for each batch are listed in Table 2.

**Figure 1:**
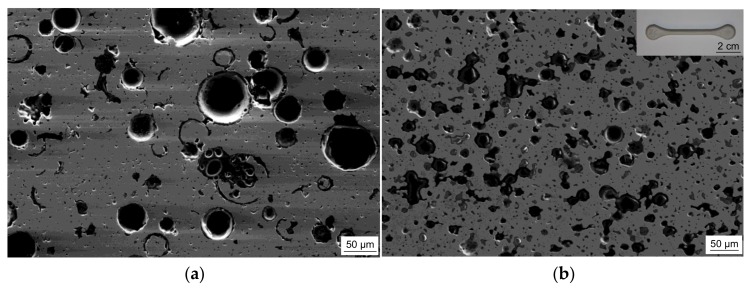
SEM images of two syntactic TRIP-steel matrix foams as-sintered: (**a**) with 40 vol % cenospheres (T4C); (**b**) containing 40 vol % glass microspheres (T4G); the overlay image in (**b**) illustrates the raw dog-bone sample.

After mixing for 1 h at 125 °C, the feedstock was filled in a Ferromac FM40 injection molding machine from (Klöckner-Ferromatic, Malterdingen, Germany). For the processing of the dog-bone shaped green parts, a feedstock temperature of 145 °C, a mold temperature of 42 °C and an injection pressure of 28 bar were applied. The binder was removed in a two-stage process starting with chemical debinding for 48 h at 40 °C in hexane followed by thermal debinding with slowly heating to 500 °C and holding for 1 h. The final pressure-less sintering was carried out equally at two temperature regimes, first for 3 h at 1000 °C and second for 1.5 h at 1200 °C. Both the green and sintered products (see overlay image in Figure 1b) were checked by means of XCT with regard to the occurrence of material inhomogeneities, such as pores or shrinkage cracks.

### 2.2. Testing Procedure and Fundamentals

First, the densities of the sintered products were measured by means of Archimedes’ principle and further verified by determining the mass-volume ratios of the compressive test specimens prepared from the sintered parts. The determination of carbon concentration was performed by analyzing low and high CO_2_ and CO gases which are produced during O_2_-carrier gas hot extraction (CGHE) in a high performance carbon/sulfur analyzer of type EMIA-820V (HORIBA Ltd., Kyoto, Japan) with a measurement accuracy of less than 0.3 ppm.

The mechanical responses of the materials were investigated by uniaxial quasi-static compression and dynamic impact testing at strain rates of $\dot{\varepsilon }=d\varepsilon /dt=0.01\;s^{-1}$ and $200\;s^{-1}$, respectively. While the quasi-static test series were carried out in a servo-hydraulic universal testing machine of type MTS 810, the dynamic behavior was recorded in an instrumented drop-weight tower using an impact mass of 180 kg. At least four samples of each material batch were tested. The crush resistance of the specimens was evaluated by calculating the 0.2% proof stress $\sigma_{0.2}$, the mean or plateau strength $\sigma_{p}$ and the specific energy absorption (SEA) per unit mass. According to the recommendations of DIN 50134 and ISO13314:2011 [37,38], the parameter $\sigma_{p}$ (for a continuous increase in stress) was determined by

(1) $$\sigma_{p}=\sigma_{40}-\frac{\sigma_{40}-\sigma_{20}}{2}$$

measuring the minimum and maximum stress related to 20% and 40% engineering strain, respectively. SEA was given by the absorbed energy of the material when achieving the assumed densification limit of $1.3\;\sigma_{p}$.

The topology and the damage phenomena of selected differently deformed syntactic foams were studied by XCT using a Phoenix nanotom^®^ m (GE, Wunstorf, Germany) at an acceleration voltage of $U_{acc}=130\;\mathrm{kV}$ with a current of I=100 µA and a geometrical magnification of about 27x resulting in a voxel size of $v_{x}=3.7\;µm$. The reconstruction, visualization and evaluation of the microtomography data were done by means of the software datos|x 2.2 (GE), VGStudio (Volume Graphics, Heidelberg, Germany) and Avizo 8 (FEI Visualization Sciences Group, Burlington, NJ, USA). Further damage features and microstructure characteristics were detected by SEM and EBSD phase measurements using a TM-1000 tabletop microscope (Hitachi, Tokyo, Japan) and a FEG-SEM LEO 1530 GEMINI (Zeiss, Oberkochen, Germany) in combination with the software HKL Channel 5, version 5.09 (Oxford Instruments, Abingdon, England).

In order to measure the content of the strain-induced $\alpha^{\prime }$-martensitic phase (ferromagnetic) after sample deformation, magnetic force balance (MB) measurements based on ferritic/martensitic calibration samples and magnetization measurements via a vibration sample magnetometer, VSM (PPMS, Quantum Design, San Diego, CA, USA) were applied. The latter was performed in a magnetic field of $\mu_{0}H=\left(-1.2\ldots 1.2\right)T$ (1 T ≅ 10000 Oe) with a frequency of 40 Hz and a peak amplitude of 2 mm.

Since the content of δ-Fe was proven to be negligibly small, the total amount of the measured ferromagnetic fraction after plastic deformation was attributed to the strain-induced $\alpha^{\prime }$-martensite formation.

The ferromagnetic martensitic phase fraction of the steel matrix $f^{\alpha^{\prime }}$ as a function of material condition c, engineering strain ε and nominal strain rate $\dot{\varepsilon }$ was determined by

(2) $$f^{\alpha^{\prime }}\left(c,\;\varepsilon ,\dot{\varepsilon }\right)=\frac{F^{*}}{F_{cal}}\cdot \frac{m_{cal}}{m_{m}^{*}}\cdot f_{cal}^{\alpha^{\prime }}$$

for the magnetic balance measurements and

(3) $$f^{\alpha^{\prime }}\left(c,\;\varepsilon ,\dot{\varepsilon }\right)=\frac{M_{s}^{*}}{M_{s}^{cal}}\cdot \frac{m_{cal}}{m_{m}^{*}}$$

for the VSM experiments. $F^{*}$, $F_{cal}$ indicate the forces to whom the specimen and the fully ferritic/martensitic calibration sample are subjected in the magnetic field and $M_{s}^{*}$, $M_{s}^{cal}$ are their intrinsic saturation magnetization values (near constant magnetization) at ±1.2 T. A saturation magnetization of $M_{s}^{cal}=155.1\;{\mathrm{Am}}^{2}/\mathrm{kg}$, measured for a forged X5CrNiMo16-5-1 steel with 100% bcc crystal structure, was used as standard material for the VSM series. This value is comparable with the experimental and numerical results for 100% martensitically transformed AISI 304 given in previous publications [39,40,41,42]. The mass of the MB calibration samples $m_{cal}$ ranged from 154 to 306 mg using a ferromagnetic phase fraction of $f_{cal}^{\alpha^{\prime }}=100\%$.

In order to exclude the content of microspheres, the mass of the transformable steel matrix $m_{m}^{*}$ was used, as listed in Table 2. Based on the assumption that the densities of the fcc austenitic and the bcc martensitic phase are nearly the same, $f_{cal}^{\alpha^{\prime }}$ can be given in vol % which is equal to the relative mass fraction.

## 3. Results

### 3.1. Characterization of the As-Sintered State

The density of the reference bulk material is 7.42 g/cm^3^ and, therefore, at about 93% of the theoretical density (see also Table 3). The densities of the foam specimens are—depending on the composition—in the range of 4.25 to 6.6 g/cm^3^. The actual foam densities are higher than the calculated theoretical values in analogy to the behavior observed with e.g., Invar foams [42]. The foams based on cenospheres show slightly higher densities as the ones with hollow glass spheres which can be attributed to uncertainties of the cenosphere’s specific density (0.600–0.850 g/cm^3^) which led to systematic deviations in the mixing procedure.

The microstructures of the sintered syntactic TRIP-steel matrix foams either containing cenospheres or glass microspheres are different. In both cases, the second component is well-dispersed within the metal matrix also containing some residual sinter porosity. As shown in Figure 1a, exemplarily for the material with 40 vol % Fillite 106, the intact cenospheres exhibit a high sphericity and a high effective pore volume. As comparable to previous investigations on Invar-based syntactic foams [43], cenospheres that are broken and/or filled by the matrix material during the synthesis process can be detected. In the case of the congeneric TRIP-steel/S60HS foams, the microspheres are less regular in shape and more clustered (see Figure 1b).

**Figure 2:**
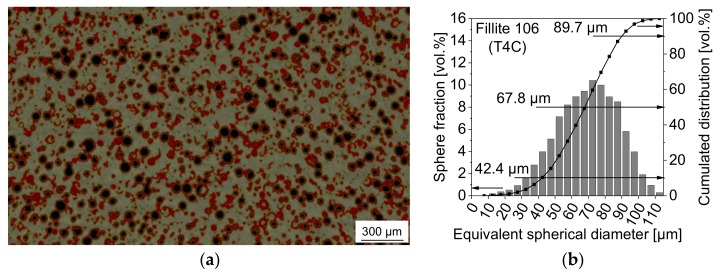
Tomographic imaging of the syntactic TRIP steel matrix foam with 40 vol % Fillite 106: (**a**) reconstructed 2D image (pale grey—matrix, cenospheres: red—solid part, black—cavities/pores); (**b**) sphere size distribution (using equivalent spherical diameter, ESD).

**Figure 3:**
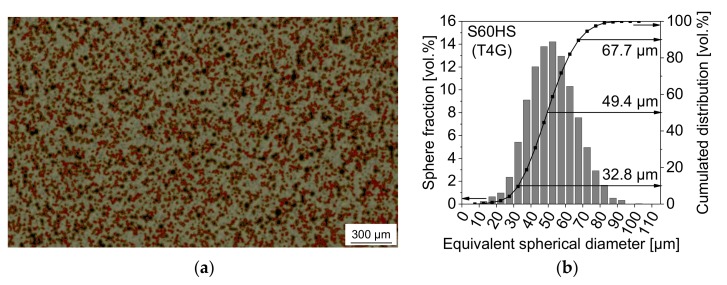
Tomographic imaging of the syntactic TRIP steel matrix foam with 40 vol % S60HS: (**a**) reconstructed 2D image (pale grey—matrix, cenospheres: red—solid part, black—cavities/pores); (**b**) sphere size distribution (using equivalent spherical diameter, ESD).

By using XCT, defined material volumes of the conditions T4C and T4G were non-destructively imaged and reconstructed in 3D. According to the principle of greyscale correlation and depending on their X-ray absorption properties, the three material components including the matrix, pores and sphere parts have been assigned to certain threshold values indicated by different color coded regions of interest (ROIs). Therefore, it was possible to visualize the microstructure features before (Figure 2a and Figure 3a) and after deformation. In order to determine the particle/sphere size distribution of the syntactic foams, the grey value segmentation was done by means of the ISO-50% method [44] and adequate visual adjustment by interpreting the dataset’s grey value histogram. In that case, pores and sphere parts were considered as one assembly or ROI. The separation of spheres from clusters was realized by applying the standard watershed algorithm of the software Avizo 8. This algorithm generally worked for most of the spheres. However, some very irregular-shaped clusters could not be separated by that method. The final sphere size is given by the Equivalent Spherical Diameter (ESD) of each regarded object which is equal to the diameter of a sphere of equivalent volume. The sphere size distribution referred to the measured volume fraction and the cumulated volume fraction of the objects with respect to the total particle volume.

As shown in Figure 2b and Figure 3b, the majority of the cenospheres of batch T4C ranges between 40 and 90 µm with *d_50_*_(vol)_ = 68 µm, whereas most of the glass microspheres of T4G are in a narrower range of 30 to 70 µm resulting in a smaller sphere size *d_50_*_(vol)_ = 49 µm (slightly overestimated because of cluster formation).

The CHGE measurements showed increased carbon contents in the sintered test specimens in comparison to the raw metal powder (see Table 3). The highest carbon content was found for the reference compact samples T0. The foam samples exhibited generally lower C-contents and the batches T4G and T6G even less carbon than the raw metal powder. As proven by additional SEM investigations, the δ-ferrite phase (also bcc and ferromagnetic) was nonexistent within the as-sintered austenitic steel matrices.

### 3.2. Mechanical Properties

**Figure 4:**
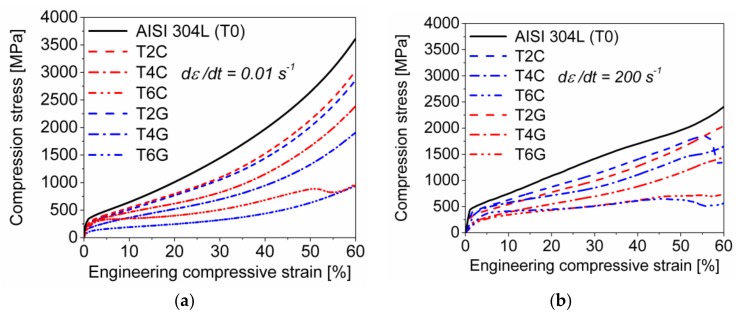
Engineering stress-strain curves of the TRIP-steel bulk specimens and the syntactic foams with S60HS glass microspheres or Fillite 106 cenospheres under (**a**) quasi-static (0.01 s^−1^) and (**b**) dynamic compressive loading (200 s^−1^).

Both the quasi-static and the dynamic compressive stress-strain responses of the syntactic TRIP-steel matrix foams (Figure 4a,b) are indicated by a quasi linear-elastic regime, a plateau region and final densification. As similar to the pure matrix material, the higher-density syntactic foams containing volume fractions of spheres of 20% and 40% exhibit a smooth elastic-plastic transition and a less pronounced plateau region with a gradual increase in stress. By contrast, an initial stress peak at the onset of plastic yielding and a flat load plateau was observed for the material with 60 vol % Fillite 106. Finally, a sharp increase in stress initiates the total densification of the foam accompanied by barreling of the sample. As expected, the mean stress level decreases with rising volume content of microspheres and porosity, respectively. The syntactic foams with 20 vol % glass microspheres or cenospheres exhibit a very similar stress-strain response, particularly controlled by the behavior of the steel matrix. However, for higher filler contents, distinctive differences between the syntactic foam batches and a strong alteration with respect to the pure TRIP steel specimens could be observed.

It is obvious that the mean compression strength of the TRIP-steel/Fillite 106 specimens with volume fractions of spheres ≥40% is significantly higher than the competing variants containing the glass microspheres. However, especially their crush resistance under dynamic impact loading is limited up to 50% compressive strain. Above that value, the foams with cenospheres failed as indicated by a stress drop.

**Figure 5:**
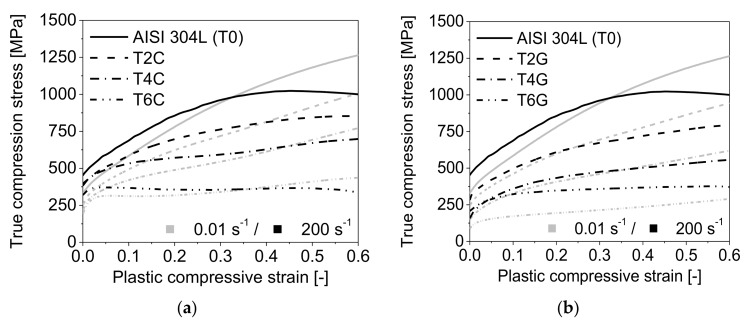
Comparison of the plastic flow behaviors of the TRIP-steel matrix material and the syntactic foams with respect to quasi-static and dynamic compressive loading: (**a**) TRIP steel/Fillite 106; (**b**) TRIP steel/S60HS batches and the pure TRIP steel (T0) as reference.

In order to demonstrate their strain rate sensitivity, the mechanical responses of the TRIP-steel syntactic foams were expressed by true stress-plastic strain curves (with beginning at the calculated 0.2% proof stress), considering the materials as porous bulk products. As clearly shown in Figure 5a,b for the entire test series, the dynamic compression stress level in the early stage of plastic deformation is significantly enhanced as compared to quasi-static loading.

The strain rate sensitivity of the materials at the onset of plastic yielding can be derived from the following relationship:

(4) $$\frac{\sigma_{0.2}{}^{\left(d\right)}}{\sigma_{0.2}{}^{\left(s\right)}}={\left(\frac{{\dot{\varepsilon }}^{\left(d\right)}}{{\dot{\varepsilon }}^{\left(s\right)}}\right)}^{m}$$

where $\sigma_{0.2}{}^{\left(d\right)}$ and $\sigma_{0.2}{}^{\left(s\right)}$ are the dynamic and quasi-static proof stresses at a plastic strain of 0.2% corresponding to the strain rates ${\dot{\varepsilon }}^{\left(d\right)}=200\;s^{-1}$ and ${\dot{\varepsilon }}^{\left(s\right)}=0.01{\;s}^{-1}$; the exponent m represents the strain rate sensitivity at the elastic-plastic transition.

The pure TRIP steel material responds to the strain rate increase of more than four orders of magnitude with a dynamic stress enhancement of ~35% which is related to a strain rate sensitivity m=0.030. The syntactic foams with cenospheres as secondary component exhibit generally a much higher but non-continuous increase in proof stress (52–75%) reflected by m-values in the range of 0.042–0.056. By contrast, the materials containing S60HS glass microspheres tend to a less pronounced strain rate effect (m=0.020 and m=0.015 for T2G and T4G, respectively), excluding the batch T6G whose proof stress was doubled specified as the highest strain rate sensitivity (m=0.071) in terms of the present test series. Luong et al. (2016) [43] calculated an exponent m of 0.020 and 0.018 for Invar-based syntactic foams with 5 and 10 vol % cenospheres, as similar to the results obtained for the materials T2G and T4G but lower than those of T2C, T4C, T6C and T6G.

However, with rising compressive deformation a distinctive alteration in the dynamic stress-strain response with regard to the quasi-static loading mode can be observed. When exceeding a critical plastic strain ≥0.1, the slope of the dynamic stress-strain curve gradually decreases and finally stagnates, thus achieving a saturation level which lies below the quasi-static curve.

**Figure 6:**
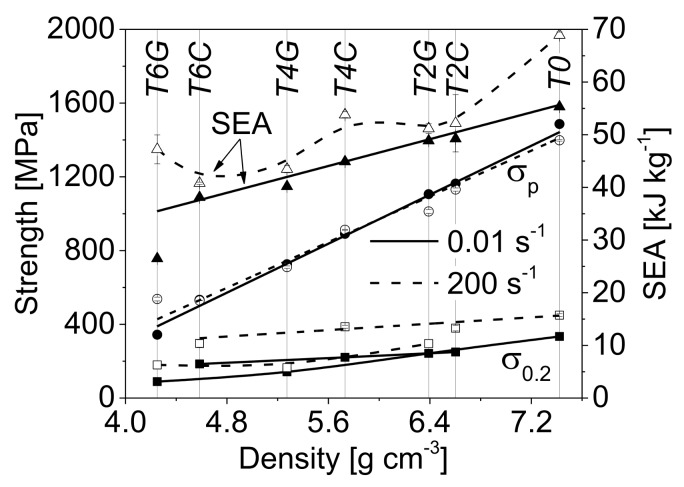
Compression strength properties and specific energy absorption of the different TRIP-steel matrix syntactic foams and the matrix reference material with respect to strain rate (0.01 s^−1^, 200 s^−1^) and density.

The effects with respect to material density, kind and volume content of spheres and strain rate on the mechanical responses of the studied TRIP-steel syntactic foams are represented by the common crush characteristics of cellular materials including the 0.2% proof stress ($\sigma_{0.2}$), the plateau strength ($\sigma_{p}$, defined by Equation (1)) and the specific energy absorption per unit mass (SEA), listed in Table 4. As expected and illustrated in Figure 6, all the parameters follow the same rising trend with increasing material density. However, the (mean) plateau strength is indicated by a quite linear but much steeper increase with density than the proof stress. It is also obvious that the plateau strength corresponding to a range of engineering strain between 20% and 40% is nearly unaffected by strain rate. Hence, the initial dynamic stress enhancement is suppressed or completely invalidated by the stress drop at higher deformation degrees.

A different evolution as a function of material density comparing quasi-static and dynamic loading was determined for the specific energy absorption. By excluding batch T6G, SEA results for the quasi-static mode reveal a linear relationship with density, whereas the values under dynamic loading follow no regular trend. The energy absorption capability of the TRIP-steel syntactic foams with Fillite 106 cenospheres was generally higher than those containing S60HS glass microspheres. A reverse finding was only noticed for the compositions with 60 vol % spheres under dynamic impact loading. In that case, the T6G foam exhibits disproportionately high plateau strength and thus higher specific energy absorption than the competing batch T6C (47.2 kJ/kg for T6G vs. 40.8 kJ/kg for T6C).

### 3.3. Microstructure Investigations

Deformation and microstructure mechanisms on different length scales contribute to the flow behavior of the syntactic TRIP-steel foams. First, the structural evolution of the spheres and their interactions with the steel matrix under compressive loading are shown based on 3D rendered XCT images and SEM failure patterns.

**Figure 7:**
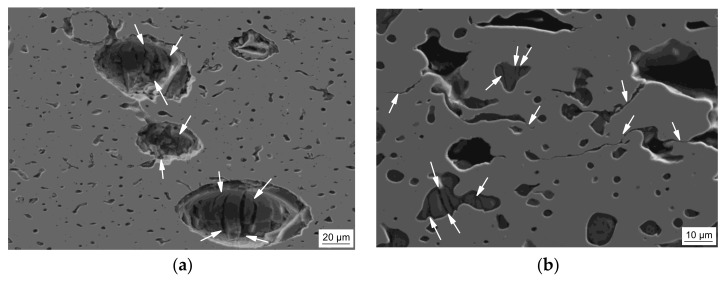
Failure patterns of the TRIP-steel syntactic foams at 20% strain under quasi-static compressive loading: (**a**) with 40 vol % Fillite 106 cenospheres (T4C); (**b**) with 40 vol % S60HS glass microspheres (T4G); the arrows mark the failure sites. (Load direction is vertical).

Both the ceramic and the glassy secondary component exhibit no noticeable structural change during elastic deformation, maintaining their as-sintered states. At the transition from the elastic regime to the plastic plateau region the failure of the spheres indicated by an inner crack formation mainly along or slightly inclined to the loading axis is initiated ([45]). With continued plastic deformation, the cracks propagate and induce the fracture of spheres (Figure 7a) and irregular inclusions (Figure 7b), respectively.

**Figure 8:**
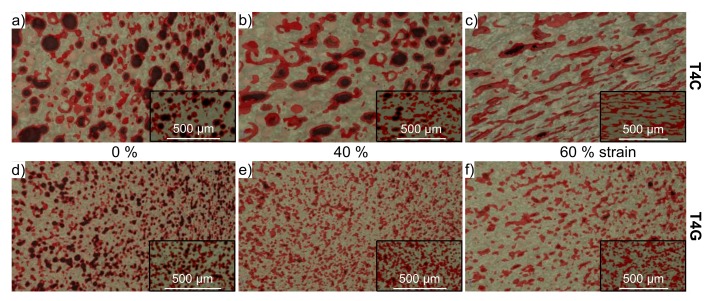
Structure evolution of the TRIP-steel syntactic foams under quasi-static compression: (**a**–**c**) with 40 vol % Fillite 106 cenospheres (T4C); (**d**–**f**) with 40 vol % S60HS glass microspheres (T4G); pale grey—matrix, red—sphere shells/inclusions, black—cavities/pores. (Load direction is vertical).

Due to shell fracture/fragmentation the cenospheres lose their initial integrity (Figure 8a), are closed and transform into an elliptical shape (Figure 8b). Finally, the barreling and densification of the T2C, T4C and T6C samples are accompanied by a rearrangement and alignment of collapsed cenospheres to chain-like clusters (“lateral extrusion” [45]) within the shear-cross region (Figure 8c).

The structural evolution of the glass-containing syntactic foams is more difficult to comprehend since only a small quantity of more or less regular spheres remained after processing (Figure 8d). These spheres are closed during plastic deformation of the sample and form finally irregular clusters or agglomerates with the neighboring inclusions (Figure 8e). A preferred orientation or even an alignment of those clusters during densification could not be proven (Figure 8f). However, a further failure mechanism, which is more pronounced for the T2G, T4G and T6G materials, is the crack propagation and crack branching through matrix ligaments originating from the glassy clusters and inclusions, as denoted in Figure 7b.

**Figure 9:**
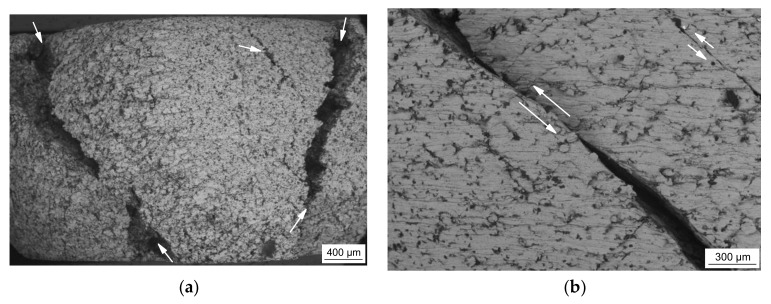
Damage of two selected TRIP-steel syntactic foams at 60% strain under dynamic impact loading: (**a**) with 60 vol % S60HS glass microspheres (T6G); (**b**) with 20 vol % Fillite 106 cenospheres (T2C); the arrows mark the damage sites. (Load direction is vertical).

Consistently, the degree of damage is enhanced and the failure is accelerated with increasing volume content of the secondary component within the steel matrix and with increasing strain rate. Hence, macro cracks originating from the free outer surface of the specimens could be detected (Figure 9a). Moreover, the formation of macro shear zones within the sample was more dominant in materials being subjected to dynamic impact loading in the drop weight tower (Figure 9b).

In the second part of this chapter, the microstructure characteristics and the phase evolution of the TRIP-steel matrix related to the kind and volume fraction of the spheres and the applied deformation degree and strain rate are shown in detail.

**Figure 10:**
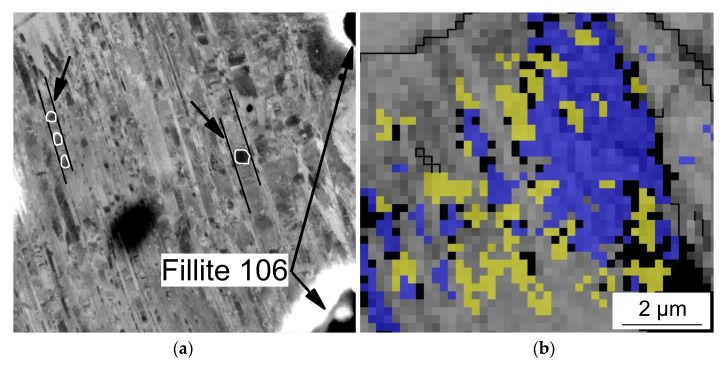
(**a**) BSE image (arrows indicate $\alpha^{\prime }$-martensite within deformation bands) and (**b**) EBSD phase map of the syntactic foam with 20 vol % Fillite 106 after 20% quasi-static deformation; for EBSD: grey—band contrast of austenite, yellow—ε-martensite, blue—$\alpha^{\prime }$-martensite, black dots—not indexed, black line—High Angle Grain Boundaries (HAGBs), coincidence site lattice CSL boundaries.

Corresponding to their transformable austenitic steel matrices, all specimens exhibited the formation of deformation bands and martensitic phases during plastic compressive deformation at room temperature. Deformation bands and local regions with hexagonal lattice structure (viz. ε-martensite) as well as bcc a'-martensite nuclei within the deformation bands were detected by high-resolution BSE imaging (Figure 10a) and EBSD measurements (Figure 10b).

Based on the results of the magnetic balance (MB) and VSM measurements, significant differences in the volume fraction of the ferromagnetic $\alpha^{\prime }$-martensite depending on material composition, (global) deformation degree and applied strain rate were observed.

**Figure 11:**
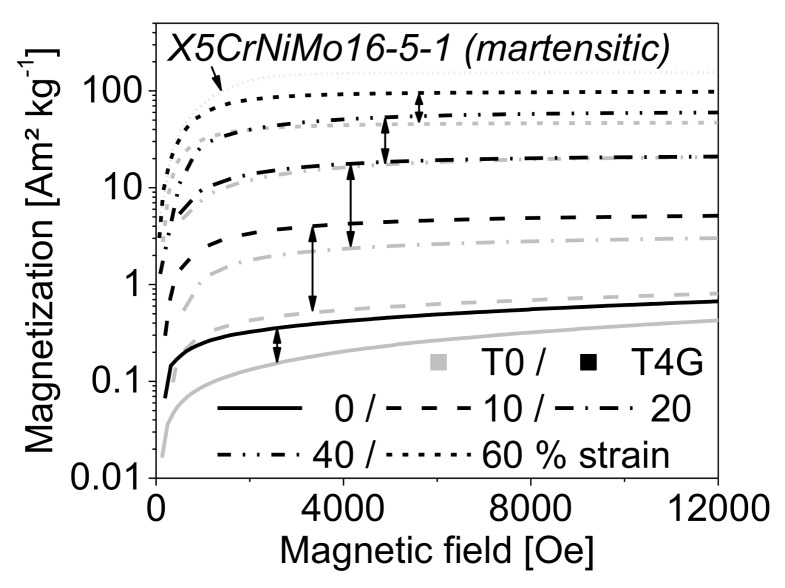
Magnetization curves of the syntactic foam with 40 vol % S60HS, the pure TRIP-steel bulk specimens and the fully martensitic reference material with regard to different deformation degrees under quasi-static loading.

In general, the increase in compressive strain was associated with a shift of the magnetization curves (viz. measured magnetization vs. magnetic field in VSM) to higher magnetization values and an enhancement of the intrinsic saturation magnetization $M_{s}^{*}$ (viz. end of plateau region at a magnetic field of 12,000 Oe (1.2 T), see Figure 11). The measured $M_{s}^{*}$-values were used to calculate the ferromagnetic volume content of each material state, according to Equation (3). The comparison of the magnetization curves of the pure steel matrix material and a selected syntactic TRIP-steel foam (here: using batch T4G as an example) illustrates that the foam samples exhibit higher magnetization levels than the reference steel bulk specimens with regard to the same engineering strain. Besides, the tested materials did not meet the saturation magnetization limit of the reference X5CrNiMo16-5-1 bulk material which represents the fully martensitic state. The highest value (~98 Am²/kg) was achieved by the syntactic foam with 40 vol % S60HS glass microspheres (T4G).

**Figure 12:**
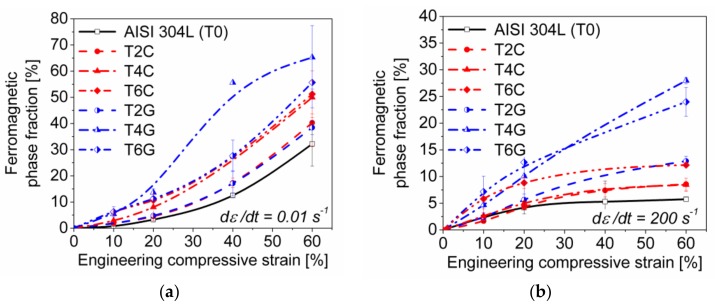
Ferromagnetic phase fractions (α’-martensite) of the TRIP-steel syntactic foams and the bulk matrix material as functions of compressive strain for (**a**) quasi-static (0.01 s^−1^) and (**b**) dynamic impact loading (200 s^−1^).

Figure 12a,b show the alteration of the (ferromagnetic) $\alpha^{\prime }$-martensite phase fraction for all studied materials by means of MB as a function of strain related to quasi-static and dynamic loading, respectively. For each material condition and deformation state a set of minimum three samples was measured whose fluctuations in phase content are represented by the calculated standard deviations.

In the case of quasi-static compression ($\dot{\varepsilon }=0.01\;s^{-1}$), the $\alpha^{\prime }$-martensite reveals a gradual increase up to ~20% strain and then a steeper increase with rising compressive deformation following a nearly exponential trend, as described by the Olson-Cohen kinetic model. By contrast, the ferromagnetic phase evolution under dynamic loading ($\dot{\varepsilon }=200\;s^{-1}$) reflects a parabolic behavior passing into a final deformation stage where the martensite content seems to stagnate at values quite below the results which were determined under quasi-static loading.

**Figure 13:**
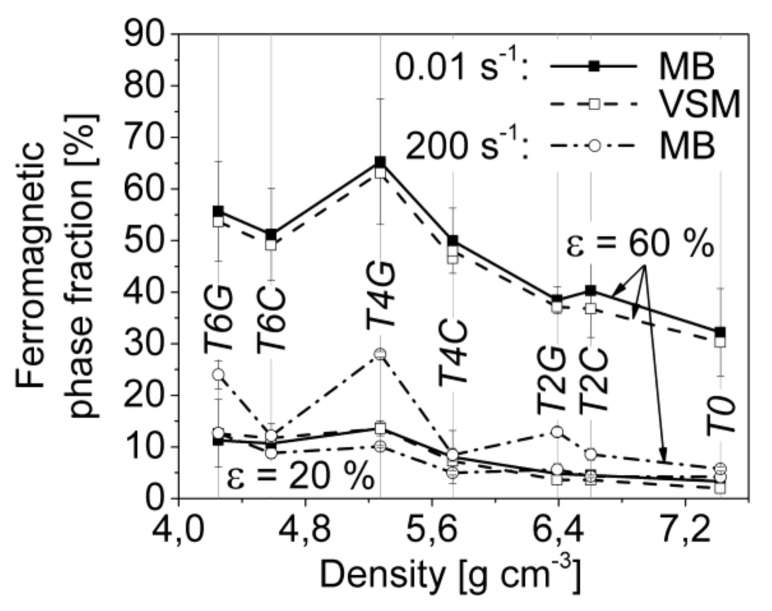
Ferromagnetic phase fraction of the different TRIP-steel syntactic foams and the matrix reference material at 20% and 60% compressive strain with respect to strain rate (0.01 s^−1^, 200 s^−1^) and density, measured by magnetic balance (MB) and VSM measurements.

**Figure 14:**
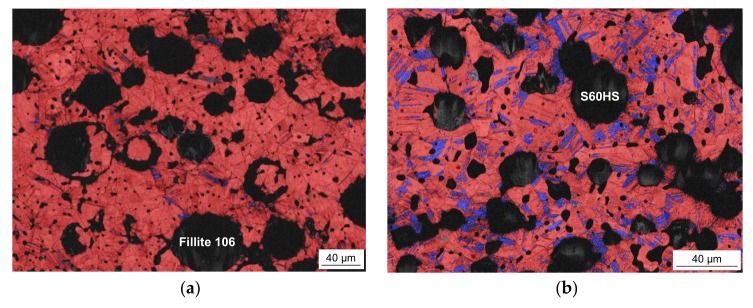
EBSD phase maps for two selected TRIP-steel syntactic foams deformed up to 10% strain under quasi-static compression: (**a**) T4C and (**b**) T4G; red—austenite (fcc), blue—$\alpha^{\prime }$-martensite (bcc), grey/black—not indexed, ε-martensite was excluded.

Figure 13 gives an overview of the ferromagnetic phase fractions corresponding to the material compositions and the loading conditions (viz. engineering strain and strain rate). The results of the magnetic balance (MB) measurements were comparable to the values obtained by the VSM experiments, which confirms the correctness of both methods. Three essential findings with regard to the strain-induced martensitic phase transformation can be pointed out: (i) the content of $\alpha^{\prime }$-martensite within the austenitic matrices of the syntactic foams is higher as compared to the pure steel bulk material (T0); (ii) the batches containing 40 and 60 vol % S60HS glass microspheres exhibit a higher martensitic phase fraction than the corresponding materials with Fillite 106 cenospheres (as proven even for low strains by EBSD measurements on T4C (Figure 14a) and T4G (Figure 14b)); (iii) the $\alpha^{\prime }$-martensite contents determined at 60% strain under quasi-static compression are a multiple of the values obtained at the same strain for dynamic impact loading (2–3 times for TRIP-steel/S60HS, 4–6 times for TRIP-steel/Fillite 106 and 5–6 times for the reference material T0).

While the pure TRIP steel achieves $\alpha^{\prime }$-martensite phase contents of 32 and 8 vol % after 60% quasi-static and dynamic deformation, respectively, volume fractions of 65% and 28% were measured for the T4G foams which were the highest for the total test series. In the case of the TRIP-steel syntactic foams with Fillite 106 cenospheres, values of 50 and 8.5 vol % for T4C as well as 51 and 12 vol % for T6C based on the two different loadings were determined.

## 4. Discussion

The measured relative density of the reference (93%) is somewhat below typical density ranges of MIM components (96% and higher). This can be explained by the sintering temperature of 1200 °C which is lower than the commonly used 1250 °C for stainless steels. 1200 °C was arbitrarily selected in order not to damage the cenospheres (1200 °C is the lower limit of the cenosphere melting range, see Table 1) while maintaining an acceptable sintering quality. The soda-lime borosilicate 3MTM microspheres and Omya Fillite^®^ FG 106 alumino-silicate cenospheres are differently affected by the processing of the syntactic foam. The latter ones exhibit a significantly higher thermal stability and thus mostly retain their initial shape after processing. In some cases broken shell fragments of FG 106 as a consequence of material processing could be observed. Possible fragmentation mechanisms can be: fracture during mixing and feedstock preparation, fracture during injection and integrity loss during sintering. In comparison, in agreement with the findings of previous publications [5,19,29,43,46], the S60HS glass microspheres become geometrically unstable at the high sintering temperature, finally melt and form both deposits at the pore surfaces and inclusions in the sintered matrix. Luong et al. [43] and Weise et al. [19] pointed out that their disintegration starts at sintering temperatures of about 1000 °C due the exceeded softening point and the decreasing viscosity of the glass. An excessive number of collapsed glass microspheres is observed when sintering at 1200 °C. Besides, those inclusions are irregular in shape and pre-cracked. The cavities are no longer fully enclosed by the glass shells. Therefore, the sphere size distribution obtained by XCT measurements does not reveal the initial sphere size of 3MTM S60HS but a disintegrated one due to processing.

The increased carbon contents especially of the batch T0 is considered to be caused by carbon entry from the binder, which consists mainly of waxes and higher melting polymers. The fact that the foams exhibit lower carbon contents than the reference bulk specimens can be explained by two potential mechanisms. At first, the gas permeability is higher in the specimens with hollow cenospheres and (collapsing) hollow glass spheres than in the reference material which consists only of very small metal powder. This allows faster transport of hydrogen as reactant and carbon-hydrogen-molecules as reaction product. Secondly, the silicate phases of the cenospheres and the glass spheres can support the decomposition of the binder and the reaction with the sintering atmosphere.

As expected, the compressive stress-strain curves of the TRIP-steel syntactic foams show the typical reduction of stress level and the formation of a plateau with increasing porosity and decreasing material density, respectively. The mechanical response of the syntactic foams is controlled to a large extent by the strength and the deformation behavior of the AISI 304L matrix governed by strain hardening, phase transformation and strain-rate sensitivity. The second contribution is performed by the resistance and breakage of the hollow particles. Obviously and as observed for the batches T2G and T2C, the differences of the sphere types are not important for the overall behavior of foams with only low contents of spheres. For higher sphere contents the situation changes and differences will become apparent.

The TRIP-steel syntactic foams reveal a smooth gradual transition from the elastic region to the plastic plateau-like region. This is contrast to other kinds of MMSFs such as those based on aluminium alloy or magnesium alloy matrices [47,48,49] which fail by a stress drop after passing a peak stress. This is a result of the reduced load bearing capacity caused by the first fracture of spheres, the crack propagation into the matrix and shear failure of the specimen [47]. In the case of stainless steel MMSFs, the crushing of the hollow particles can be more compensated by the matrix because of its high ductility and fracture toughness as compared to Al alloys [50].

Similar to other MMSFs with comparably high specific densities [16,17,18,29,43], regions of the TRIP-steel AISI 304L syntactic foams are less pronounced and more dominated by a gradual increase in compression stress. The tendency to form a stress plateau increases with reducing density and rising volume content of the hollow particles because deformation mechanisms like lamellae and strut buckling, lamellae and strut tearing and layered densification will be more pronounced for foams with higher porosities. Even if the embedded hollow spheres will influence all those local processes this will not impede the general trend. Besides the changing of the foam structure due to the progressing compression also significant strain hardening effects of the steel matrix will occur locally and influence the local and overall foam deformation behavior.

The compression stress level and the energy absorption capability of the TRIP-steel SFs with Fillite 106 cenospheres are generally higher than the values of variants containing S60HS glass microspheres which is comparable to the finding for AISI 316L steel SFs [29] but reverse to the behavior of Invar-based SFs [43]. This can be explained by the different thermal and mechanical stability of the spheres and the different sintering temperatures for the stainless steels 304L and 316L, and for Invar. In the case of the latter a sintering temperature of 1000 °C is used at which the high-strength glass spheres (strength predicted to be 124.1 MPa for the spheres [35] and 3500 MPa for the bulk material [51]) deform slightly but remain largely intact. Therefore, the spheres can influence the deformation of the metal structure and contribute to the deformation resistance of the overall structure. The Fillite exhibits higher thermal but less mechanical stability and will contribute less in this case to the overall foam strength. The situation reverses in the case of the stainless steel foams sintered at 1200 °C. Here the glass spheres have mainly collapsed and cannot contribute directly to the structure strength. In contrast, the high number of intact cenospheres offers a sufficient load transfer and effectively constrain the plastic deformation of the surrounding metal matrix [5]. The enhanced crush resistance of the batches T4C and T6C is also supported by their macro hardness which is slightly higher as compared to T4G and T6G [5].

**Figure 15:**
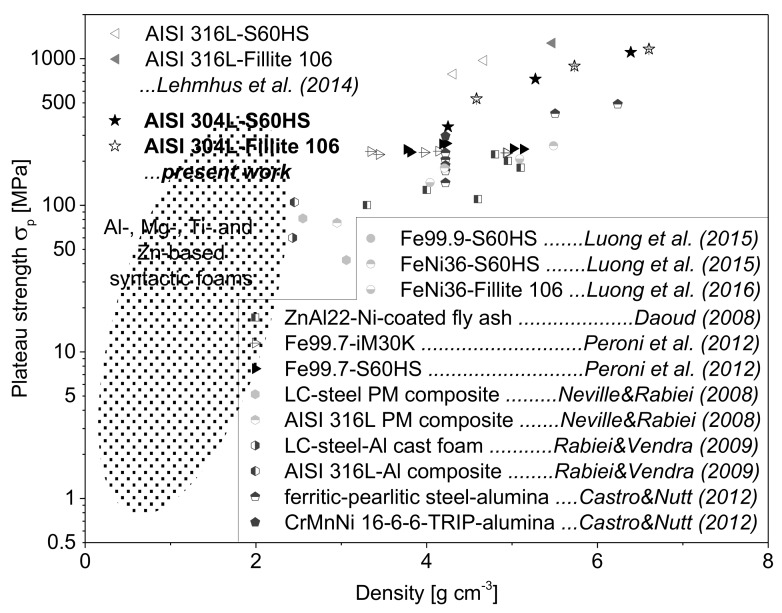
Comparison of the (mean) plateau strength and of different syntactic metallic foams under quasi-static loading, noticing that the AISI 316L- and the present AISI 304L-based foams exhibit the highest $\sigma_{p}$-values for higher densities >4 g/cm^3^; the ellipsoid strength region was extrapolated from literature [1,8,12,22,28,53,54,55,60,61,62].

**Figure 16:**
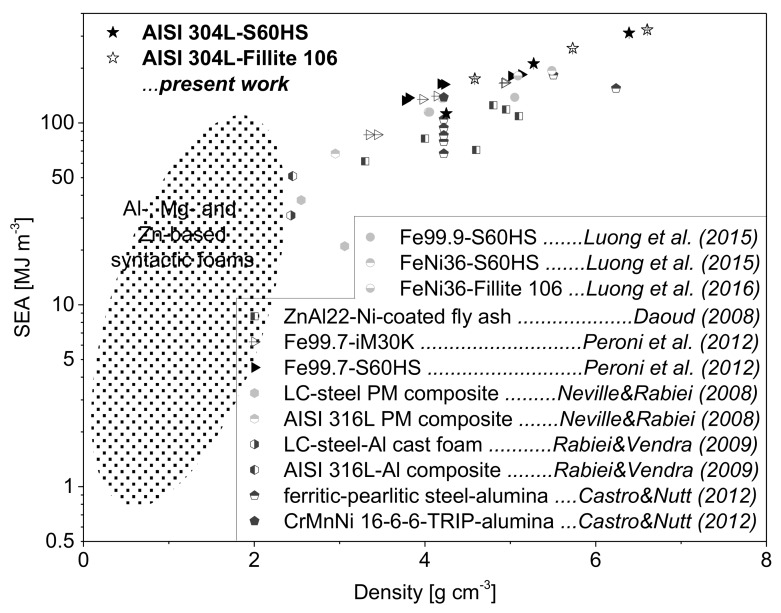
Comparison of the specific energy absorption (SEA) of different syntactic metallic foams under quasi-static loading; the ellipsoid strength region was extrapolated from literature [1,8,12,22,28,53,54,55,60,61,62].

The property charts in Figure 15 and Figure 16 give an evaluation of the plateau strength and specific energy absorption (SEA, in MJ/m^3^) of the presently studied syntactic foams in comparison with corresponding results for other types of MMSFs. The plateau strengths of the AISI 304L-SFs are lower than the values obtained for the AISI 316L-based variants (without TRIP effect) which were subject to the same conditions of the MIM process, volume contents and types of spheres [29]. This fact can be mainly attributed to the higher basic strength of the AISI 316L-steel matrix ($R_{p0.2}=359\;\mathrm{MPa}$, UTS=726 MPa [29]) as compared to the AISI 304L bulk material ($R_{p0.2}=259\;\mathrm{MPa}$, UTS=570 MPa [5]) determined by quasi-static tensile tests. However, in general the materials with strain hardening austenitic stainless steel matrices surpass the common Al-, Mg-, Ti-, Zn-, Invar- and pure Fe-based syntactic foams [1,8,12,16,22,28,43,51,52,53,54,55,56,57,58,59,60,61,62] as well as other steel composite foams [18,63,64].

The mechanical response of the TRIP-steel syntactic foams with increasing nominal strain rate is characterized by an initial dynamic strengthening and a final stagnation in compression stress, mainly controlled by the intrinsic strain-rate sensitivity of the corresponding matrix material. The strengthening in the early stage of plastic deformation (here: measured stress at 5% engineering strain) manifests by a stress increase of 12–25% for the cenosphere-variants, as similar to the AISI 304L bulk material (~22%) and also comparable to the results obtained for the AISI 316L/Fillite 106 syntactic foams (20–25%) [29,46]. In comparison, the materials T2G and T4G exhibit a dynamic strength enhancement of only ~11% and ~8%, respectively.

The strain hardening of the compact 304L reference material is governed by the generation of dislocations and stacking faults and the activation of glide systems in favorably oriented austenitic grains preferably near high-angle grain boundaries (HAGBs) and in the immediate vicinity of matrix/sphere interfaces [5]. Besides, the strain-induced phase transformation to $\alpha^{\prime }$-martensite (obviously following the $\gamma \to \varepsilon \to \alpha^{\prime }$ sequence) within and at the crossing points of deformation bands makes a further essential contribution to the mechanical response. For the foams the failure of the spheres or inclusions and the interaction of spheres and matrix have to be considered. The microstructural defects form in favorably oriented austenitic grains preferably near High-Angle Grain Boundaries (HAGBs) and steel/particle interfaces. At the interfaces perpendicular to the loading direction an increased plastic strain with enhanced tendency for the generation of $\alpha^{\prime }$-martensite nucleation sites is expected ([65]).

The significant decrease of the strain hardening rate when exceeding a certain plastic deformation degree can be attributed to two factors: the quasi-adiabatic heating of the specimen due to the resulting deformation heat which remains to a great magnitude (~95%) in the steel matrix and the strain rate decline during the drop weight impact test. In agreement with previous findings [32,33,46], the deformation temperature in cellular austenitic stainless steel structures being subject to strain rates of 10^2^–10^3^ s^−1^ can go up to 100 °C (or temperature gradient of ~80 K) or even higher when the failure is localized in shear bands [66]. Batches with higher density as the reference T0 exhibit higher deformation stresses and, hence, higher deformation work. On the other hand their thermal mass is also higher. Therefore, adiabatic softening effects should be perceptible for all tested materials. Indeed, for all batches with the exception of T6G the curves for the true stress for high-rate compression drop below those of the corresponding quasi-static tests. Furthermore, the relation of the true quasi-static and dynamic compression stresses at a strain of 0.6 is between 1.2 and 1.3 for all batches.

Shear band formation at high strain rates can be triggered by the collapse of the sphere particles because they fracture more easily under shear as compared to compression [48,51,67], also promoted by the accelerated deformation. This enhanced degree of damage under dynamic impact loading can—in addition to the thermal softening effect and gradual decrease in strain rate—be responsible for the decrease in the compression stress level at higher strains. Consistently, the stress decline is most pronounced for the pure steel bulk material and weakens with decreasing volume of matrix and increasing volume fraction of spheres, respectively. It cannot be excluded that the strain rate hardening as a result of constraining effects of the hollow particles/inclusions ([68,69]) or even micro-inertia effects [20] contribute to balance partially the material softening.

As already known, the $\alpha^{\prime }$-martensite kinetic described by its nucleation rate and volume fraction depends significantly on the applied strain rate and the arising deformation temperature. Since the test conditions under dynamic impact loading and higher plastic strains change from isothermal to quasi-adiabatic, the strain-induced martensitic phase transformation starts to stagnate. When exceeding a critical temperature threshold, the movement and formation of dislocations and stacking faults are preferred to $\alpha^{\prime }$-martensite nucleation and growth. The reduction in $\alpha^{\prime }$-martensite and the increase of thermally-activated dislocation glide mechanisms cause the observed material softening in the stress-strain curves ([32,33]). The fact that changing the strain rate leads to shifts of the contributions of the various deformation mechanisms is highlighted by the measurements of the ferromagnetic phase fraction. Those results show that deformation with high strain rates lead at first (strains < 30%) to higher ferromagnetic phase fractions, i.e., more intensive martensite formation. The situation changes for higher strains, when effects like adiabatic heating should begin to have an effect: more martensite can be found in the quasi-static specimens.

As already discussed the progressive deformation leads to a gradual increase in the ferromagnetic $\alpha^{\prime }$-martensite phase fraction. However, with regard to material composition, different $\alpha^{\prime }$-martensite contents were achieved. Hence, the TRIP-steel syntactic foams with S60HS glass microspheres exhibit higher values than the cenosphere-containing SFs and the pure TRIP steel bulk specimens. This phenomenon might be explained by a combination of two effects: (i) differences in matrix composition and (ii) lower constraints against matrix deformation originating from the glass microspheres/inclusions.

Regarding (effect i): An increased carbon content (under otherwise same composition) causes an enhancement of the thermodynamic austenite stability [70] and the intrinsic stacking fault energy [71], whereby the probability for a strain-induced martensitic phase transformation is reduced [72] but the matrix hardness increases. The highest C content (0.067 wt %) and consequently the lowest $\alpha^{\prime }$-martensite phase fraction were measured for the reference material T0. The lower C-content in the batches with glass spheres will in comparison favor the martensitic formation.

With regard to the mechanical response (effect ii), the disintegrated glass particles perform a low crush resistance and less constraint against the deformation of the matrix. As a consequence, the austenitic grains may more easily deform achieving high plastic strain levels which are necessary for the (strain-induced) formation and growth of $\alpha^{\prime }$-martensite. By contrast, the Fillite 106 cenospheres act as rigid obstacles which inhibit an easy plastic flow of the austenitic grains ([65]). Before the cenospheres get instable, the surrounding steel matrix is forced to a distinctive dislocation hardening thus the $\alpha^{\prime }$-martensite nucleation is suppressed or shifted to higher strain levels.

The contrary behavior of batch T6G under dynamic impact loading associated with a disproportionately high strength and specific energy absorption over a wide range of deformation might be explained by structural changes of the glass microspheres. T6G is the batch in which the volume amount of disintegrated glass sphere is the highest, leading to a high amount of small glass inclusions in the matrix. Therefore, the material structure will come closer to typical MMC’s which will lead to the generation of additional strain-rate hardening or higher-energy dissipating damage events.

## 5. Conclusions

The present study dealt with the investigation of AISI 304L TRIP-steel syntactic foams containing soda-lime borosilicate glass microspheres or alumino-silicate cenospheres and focuses on their role on the crush resistance and microstructural characteristics of the foam. Apart from the material composition, the change in loading conditions (viz. strain rate) significantly affects the mechanical and structural response of the MMSFs. The main results and findings of this research work can be summarized as follows:

(i) While the Fillite 106 cenospheres retain their initial shape, the S60HS glass microspheres undergo disintegration during processing accompanied with the formation of pre-cracked irregular glass inclusions and occurring chemical reactions with the surrounding steel matrix. Only the usage of cenospheres led to a strictly syntactic foam structure.
(ii) The stress-strain response of the TRIP-steel syntactic foams under compression is indicated by a linear-elastic regime, a smooth transition to plastic yielding, a plateau-like region with a gradual increase in stress and a region of structure densification. Their mean stress level decreases with rising volume content of microspheres and porosity. Since the cenospheres offer an enhanced resistance against the plastic flow of the steel matrix, the AISI304L/Fillite 106 SFs exhibit a higher mean stress level (expressed by the yield stress and the plateau strength) and a higher energy absorption capability than the S60HS-containing variants.
(iii) The deformation and failure behavior of the TRIP-steel syntactic foams is controlled by hardening/softening mechanisms and the strain-induced martensitic phase transformation (TRIP effect) of the austenitic steel matrix as well as the failure of the hollow particles or inclusions. Visible cracks within the secondary component were formed when achieving the elastic-plastic transition. At high strain levels collapsed cenospheres were rearranged and aligned to chain-like clusters within the shear-cross region of the specimen. Broken S60HS glass spheres and inclusions acted as initiators for matrix crack formation.
(iv) Dynamic impact compression of the MMSFs causes an initial strengthening followed by softening of the steel matrix (main factor = strain-rate sensitivity of the matrix) at higher strains as a consequence of quasi-adiabatic heating, the gradual decrease in strain rate during plastic deformation and the enhanced degree of damage.
(v) The $\alpha^{\prime }$-martensite phase evolution is affected by material composition, strain rate and deformation temperature. The highest ferromagnetic $\alpha^{\prime }$-martensite phase fractions were determined for the AISI 304L/S60HS SFs and the lowest for the pure TRIP-steel bulk material. Differences in matrix composition (varying C content), chemical reactions between S60HS particles and the steel matrix, and lower constraints against matrix deformation originating from the glass microspheres/inclusions should be the reasons for this behavior. The quasi-adiabatic sample heating under dynamic impact loading and at high strains initiates thermally-activated dislocation glide processes within the matrix which are finally preferred to $\alpha^{\prime }$-martensite formation.

The present study provides essential information about the characteristics of high-density TRIP-steel syntactic foams and their potential for future applications. The crashworthiness and energy absorption capability of steel-based SFs might be improved by using micro-scale filler materials which combine high thermal stability with high isostatic crush strength.

## Figures and Tables

**Table 1:** Properties of 3M^TM^ S60HS glass microspheres ([35] and direct information of 3M) and Omya Fillite^®^ 106 cenospheres [36].

Material Property	3M^TM^ S60HS	Omya Fillite^®^ FG 106
Sphere density (g·cm^−3^)	0.570–0.630	0.600–0.850
Sphere size (µm)	15 ($d_{10}$), 30 ($d_{50}$), 50 ($d_{90}$)	5.0–106
Transient temperature (°C)	600 (softening point)	1200–1350 (melting point)
Isostatic crush strength (bar)	1241	103–207
Component elements of shell	SiO_2_ (70–80%), B_2_O_3_ (2–6%), Na_2_O (3–8%), CaO (8–15%)	Al_2_O_3_ (27–33%), SiO_2_ (55–65%), Fe_2_O_3_ (≤6%)

**Table 2:** Compositions of the material batches (in vol % and wt %).

	AISI 304L	AISI 304L/S60HS			AISI 304L/Fillite 106		
	T0	T2G	T4G	T6G	T2C	T4C	T6C
vol % spheres	0	20	40	60	20	40	60
wt % spheres	0	1.84	4.76	10.11	1.90	4.91	10.41
wt % matrix	100	98.16	95.24	89.89	98.10	95.09	89.59

**Table 3:** Results of the density measurements and of the chemical analysis of the raw metal powder and the sintered test specimens.

Batch	Density/g·cm^−3^	C-Content in wt %
304L-powder	-	0.024 *
T0	7.42	0.067
T2C	6.60	-
T4C	5.73	0.042
T6C	4.58	0.023
T2G	6.39	-
T4G	5.27	0.013
T6G	4.25	0.015

* corresponding to manufacturer.

**Table 4:** Mechanical properties of the TRIP-steel matrix syntactic foams and the bulk matrix material under quasi-static and dynamic compression.

Batch	Strain Rate/s^−1^	$\sigma_{0.2}$/MPa		$\sigma_{p}$/MPa		SEA/kJ·kg^−1^	
T0	0.01 200	334 450	±3 ±9	1487 1398	±15 ±4	55.3 68.9	±0.1 ±0.6
T2C	0.01 200	250 379	±2 ±15	1163 1131	±3 ±9	49.2 52.2	±0.4 ±5.4
T4C	0.01 200	221 387	±8 ±8	891 913	±5 ±2	44.9 53.8	±0.1 ±0.6
T6C	0.01 200	185 296	±5 ±20	533 529	±11 ±1	38.1 40.8	±0.1 ±0.2
T2G	0.01 200	242 295	±3 ±11	1106 1012	±4 ±10	48.8 51.2	±0.2 ±0.9
T4G	0.01 200	142 165	±4 ±6	726 710	±7 ±6	40.2 43.5	±0.3 ±0.7
T6G	0.01 200	89 180	±8 ±21	344 537	±12 ±10	26.5 47.2	±0.4 ±2.7
